# Acoustic Coordinated Reset Neuromodulation: A Systematic Review of a Novel Therapy for Tinnitus

**DOI:** 10.3389/fneur.2017.00036

**Published:** 2017-02-13

**Authors:** Marie Wegger, Therese Ovesen, Dalia Gustaityte Larsen

**Affiliations:** ^1^Department of Clinical Medicine, Aarhus University, Aarhus, Denmark; ^2^Department of Otorhinolaryngology, Holstebro Regional Hospital, Holstebro, Denmark; ^3^Department of Otorhinolaryngology, Aarhus University Hospital, Aarhus, Denmark

**Keywords:** acoustic coordinated reset neuromodulation, desynchronizing, anti-kindling, tinnitus, systematic review

## Abstract

**Background:**

There are growing technological advances in the development of sound-based methods for the treatment of tinnitus. Most of these methods intend to affect the speculated underlying neurological causes of tinnitus. Acoustic coordinated reset (CR) neuromodulation is one of them. A novel method that as of yet seems inadequately reviewed.

**Purpose:**

To evaluate the current evidence on acoustic CR neuromodulation as a method for the treatment of tinnitus and to assess whether the method can be implemented in daily clinical practice.

**Methods:**

A systematic literature search was performed in 13 databases in the period from February 1, 2015 to May 1, 2016. Studies regarding acoustic CR neuromodulation as a treatment method for tinnitus were included in the present review.

**Results:**

A total of 8 studies were eligible for being reviewed comprising a total of 329 patients. Overall, the evidence level of the published literature was low. The main findings in the included studies were that acoustic CR neuromodulation was safe and well tolerated and most patients reported reduction of tinnitus symptoms. The neurophysiological basis of the method was claimed to be desynchronization, anti-kindling, and change of abnormal frequency couplings in a widespread tinnitus network comprising both auditory and non/auditory brain areas based on EEG analyses.

**Conclusion:**

The available evidence is insufficient for clinical implementation of acoustic CR neuromodulation. The limited level of evidence suggests that acoustic CR neuromodulation may have positive effects on tinnitus symptoms. Preliminary electroencephalographic data are compatible with the claim that tinnitus reduction after CR treatment is mediated by a desynchronizing effect. However, a proof for this claim is still lacking.

## Introduction

Subjective tinnitus (ST) is an auditory phantom phenomenon, where an auditory perception is not related to a physical, external or internal, and sound source (1, 2). Approximately 10–15% of the adult population experience tinnitus (3). Tinnitus occurs in varying degrees and may have different etiologies (2). Up to 85% of tinnitus cases are accompanied by hearing loss related to external noise trauma (4). Other risk factors include longevity, ototoxic medication, otological diseases, head injury, cerebral diseases, and mandibular joint disorders (5, 6).

There are several theories regarding the mechanisms of ST generation such as phantom auditory perception (7), stimulated acoustic emissions (8), the dorsal cochlear nucleus hypothesis (9–11), and increased spontaneous firing rate (SFR) (12, 13). Tinnitus is most frequently related to damage of the peripheral hearing system, thereby leading to a deafferentiation of neurons influencing more central parts of the auditory system. One of the tinnitus generation theories is that cochlear damage results in deafferentiation-induced cortical map reorganization (1). When central auditory neurons are deprived of their normal input, they begin to show responsiveness to the characteristic frequency of neighboring, less affected regions in the tonotopic map (14). Also included among the neuronal changes are increased SFRs, abnormal synaptic connectivity, and synchronization (15).

The varying causes of tinnitus generation have led to development of a wide variety of treatments. Several methods have been studied, although the benefit has not been conclusively demonstrated. The efficacy of cognitive behavioral therapy, however, is well established, and studies have shown a beneficial effect on tinnitus distress.^1^ Other methods include medical treatments (e.g., intravenous Lidocaine) and psychological approaches in combination with therapeutic sound, such as tinnitus retraining therapy. Sound therapies alone include the use of music, white noise generators, and hearing aids (16). Potential mechanisms through which sound therapy may act involve masking, habituation, and reversing of cortical reorganization through lateral inhibition.

There are growing technological advances in sound-based approaches of which the majority is designed to target the maladaptive plasticity perceived as the underlying mechanism of ST (16). Acoustic coordinated reset (CR) neuromodulation^®^ is one of them (6, 14).

The concept of CR neuromodulation was initially developed as a treatment method for Parkinson’s disease, where pathological neural synchronization was counteracted by desynchronizing, electrical deep brain stimulation. Since the inventors considered the pathological nerve activity of both Parkinson’s and ST to be characterized by hyperactivity and pathological synchronization, acoustic CR neuromodulation was introduced as a non-invasive counterpart (14).

Coordinated reset neuromodulation has been examined in several animal (17, 18) and human studies (19). Over the past few years, studies regarding the effects and mechanisms of acoustic CR neuromodulation have also been presented.

Acoustic CR neuromodulation is a patterned stimulation with tones adjusted to the patient’s dominant tinnitus frequency, which aims at counteracting pathological neuronal synchronization. Phase reset is proposed to be achieved by a repetitive stimulus delivery of tones with different frequencies gathered around the dominant tinnitus pitch (2).

**Figure 1:**
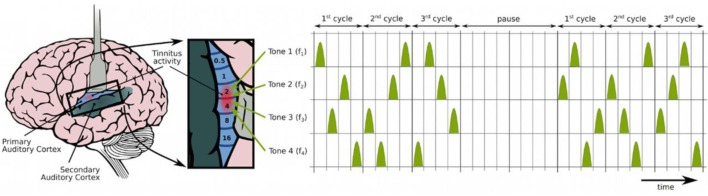
**Acoustic coordinated reset neuromodulation: the spatiotemporal organization of stimuli (23)**. The figure shows how stimuli are comprised by four different tones (triangles) gathered around the dominant tinnitus pitch in a randomized sequence during three cycles, followed by two silent cycles in a 3:2 ON–OFF pattern, to ideally enhance the desynchronizing effect (20, 24). Adapted from Chittka and Brockmann (25), with permission from Tass et al.

To facilitate understanding of CR neuromodulation in general, some principles should be addressed (Figure 1):

1. CR neuromodulation (Figure 1) is based on the phase reset of oscillatory neuronal activity through desynchronization. It is proposed to counteract the deafferentiation-induced upregulated synchrony and connectivity through desynchronizing and anti-kindling effects (20, 21).
2. Neural populations display spike timing-dependent plasticity (STDP), i.e., they continuously regulate the strength of their synaptic connectivity relative to the pre- and postsynaptic firing (spikes). In other words, neural activity and the strength of their connections are related.
3. CR neuromodulation is designed to cause long-lasting anti-kindling effects; desynchronizing stimulation of neuronal populations causes the neurons to unlearn their pathological connectivity. The method attempts to change spike firing timing networks using series of tones and to force firing from a pathological state with abnormally synchronized synapses to a desynchronized state with weaker synapses. It achieves this through tones played through hearing aid style earphones. The patients are supposed to listen to the tones 4–6 h per day for, e.g., 12 weeks.
4. The phenomenon when such states are present at the same time is known as STDP-induced multistability (2, 22).

The present review provides a systematic overview of studies of acoustic CR neuromodulation as a novel treatment method for ST. The purpose was to establish the current level of evidence available for the intervention and to assess whether the method can be recommended in the clinical setting.

## Methods

Literature search was performed in 13 databases in the period from 1st February to 1st May 2016, using the following search strings: (i) “Tinnitus” AND “acoustic coordinated reset neuromodulation,” (ii) Tinnitus AND acoustic coordinated reset neuromodulation, (iii) Tinnitus AND acoustic CR neuromodulation, and (iv) Tinnitus and acoustic coordinated reset (CR) neuromodulation. MeSH terms were not available for either tinnitus or neuromodulation. The highest number of search results was found using search string (ii). None of the other search strings resulted in additional articles. The reference lists of articles identified by this search strategy were reviewed, and one article was considered relevant and selected (26). Trial registers were also searched. A complete list of searched resources is included in Table 1.

**Table 1:** **Resources searched in following databases**.

Resources	No. hits
PubMed	14
Embase	18
Scopus	14
Web of Science	9
SveMed+	0
Cochrane	8
TRIP database	9
ProQuest	22
EBSCOhost	7
Biotechnology research abstract	1
BMJ journal	0
BIBSYS (Oria.no)	1
AMED alternative medicine	0
Others	
http://Clinicaltrials.gov	3
http://Controlled-trials.com	0
http://Clinicaltrialsregister.eu	0

The present systematic review was intended to follow the PRISMA 2009 guideline. No case control studies could be found, and only one randomized controlled trial (RCT) was available. Therefore, with regards to inclusion and exclusion criteria no restrictions were placed on study design, sample size, or date of release. Study language was restricted to English, German, Danish, Norwegian, or Swedish. Unpublished data were excluded from the review. The intention was to evaluate all studies of patients suffering from subjective tonal tinnitus treated with acoustic CR neuromodulation. Hence, the *a posteriori* inclusion criteria for suitable articles were any original research article concerning tinnitus and acoustic CR neuromodulation, its effect, and/or mechanisms of action. All articles were read by all the three authors.

The following outcomes parameters were identified: visual analog scale (VAS) score (VAS-L: loudness and VAS-A: annoyance), Tinnitus Handicap Inventory, Tinnitus Questionnaire (TQ), Tinnitus Handicap Questionnaire (THQ), The World Health Organization Quality of Life-BREF, tinnitus frequency, spontaneous EEG analysis [including changes regarding connectivity and cross-frequency coupling (CFC) in the tinnitus network], tinnitus pitch change, Tinnitus Functional Index questionnaire, TQ (TBF-12), Global Clinical Improvement-Impression Scale (CGI-I7), and Numeric Rating Scale.

## Results

**Figure 2:**
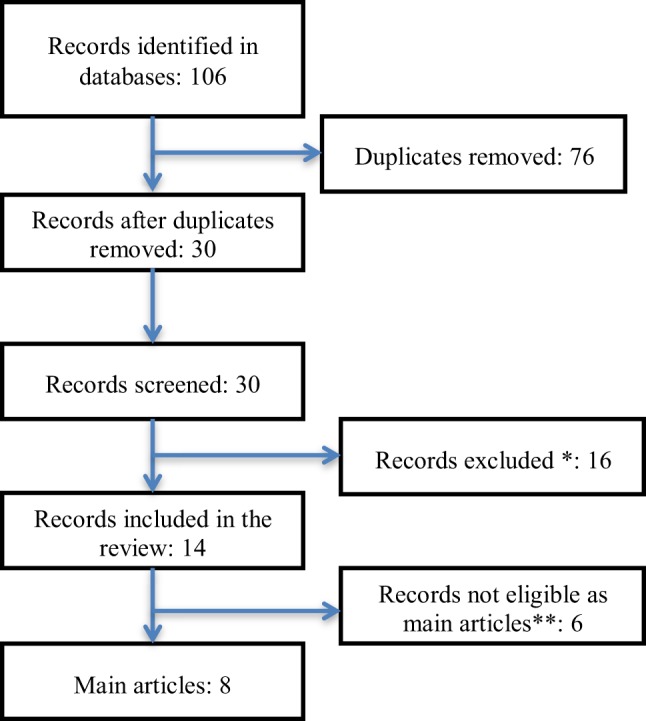
**Flowchart of the study selection**. *Abstracts, conference–proceedings, reviews, editorials, dissertations and theses, and records not on subject. **Articles assessing endpoints, Hungarian article (language restrictions), not published data, and letters.

A total of 8 publications were eligible for the review comprising a total of 329 patients in 3 main study populations (Figure 2). Details with regard to study population, design, and outcome measures are listed in Table 2. Due to the low number of studies, heterogeneous designs, overlap between study populations, and incomparability, each of the studies is commented in the following.

**Table 2:** **Overview of the eight studies included in the review**.

Reference, country	Study design	Sample	Outcome measures	Main results
Tass et al. (27), Germany	Computer analysis	None	Coordinated reset (CR) neuromodulation: model presentation illustrating the concept of CR in a simplified neuronal model, considering neurons with spike timing-dependent plasticity transformation of the concept of deep brain stimulation into non-invasive, acoustic CR stimulation	Non-invasive acoustic CR neuromodulation may be a novel therapy for tinnitus

Tass et al. (23), Germany	Prospective, randomized, single blind, placebo-controlled trial: RESET	63	Visual analog scale (VAS)	CR neuromodulation caused a significant decrease of tinnitus loudness and symptoms, and reversed tinnitus-related EEG alterations
			Tinnitus Questionnaire (TQ)	
			Tinnitus frequency	
			Spontaneous EEG	

Adamchic et al. (28), Germany	Part of RESET	59	Tinnitus pitch change versus tinnitus loudness and/or annoyance (VAS score)	VAS scores significantly correlated with the absolute value of the CR neuromodulation-induced tinnitus pitch change (*r* = 0.92 baseline to 12 weeks, *p* < 0.01)
			Changes of brain synchrony induced by CR neuromodulation versus tinnitus pitch change	Significant changes in brain activity were associated with a pronounced tinnitus pitch change

Adamchic et al. (29), Germany	Part of RESET	28	EEG pattern in the tinnitus patients after CR neuromodulation versus EEG pattern in healthy controls	Tinnitus patients significantly deviated from healthy controls concerning oscillatory brain activity
			EEG in tinnitus patients before and after acoustic CR neuromodulation	CR neuromodulation significantly normalized patient’s brain oscillations in all frequency bands
			Relationship between CR neuromodulation-induced changes of different resting EEG parameters and tinnitus symptoms	CR neuromodulation-induced normalization of EEG power was significantly associated with reduction of tinnitus severity

Silchenko et al. (30), Germany	Part of RESET	28	Comparison of EEG in tinnitus patients before and after CR neuromodulation	CR neuromodulation significantly normalized both power and causal interactions within a tinnitus-related network
			Comparison of EEG in tinnitus patients with healthy controls	CR neuromodulation specifically counteracted an imbalance of excitation and inhibition in tinnitus patients
				CR neuromodulation qualitatively changed the spectral response of the tinnitus network by modifying the shape of the averaged transfer function, so that the latter became similar to the control group

Adamchic et al. (31), Germany, USA	Re-analysis of existing dataset from RESET	59	To investigate how the oscillations in the various frequency bands interact	Identification of changes of cross-frequency coupling (CFC)
				Phase–amplitude CFC increased in tinnitus patients within the auditory cortex and the dorsolateral prefrontal regions between the phase of delta-theta and the amplitude of gamma oscillations
				Theta phase in the anterior cingulate region modulated gamma in the auditory and dorsolateral prefrontal regions

Hauptmann et al. (26), Germany, UK, USA	Prospective open-label, non-randomized, non-controlled multicenter clinical study	200	TQ (TBF-12)	TBF-12 (total score) showed a mean reduction of 4.1 points (−37.9%) compared to baseline (*p* < 0.01)
		23 study centers	Global Clinical Improvement-Impression Scale (CGI-I7)	CGI-I7 revealed that 66.9% of the patients reported an improvement of tinnitus [very much improved (8.7%), much improved (25%), or slightly improved (33.2%)] (*p* < 0.01)
			Numeric Rating Scale (NRS) (0–100)	Tinnitus-related loudness and annoyance were reduced by 11.1 points (18.9%) and 14.7 points (25.2%), respectively, compared to baseline (*p* < 0.01) on the NRS

Williams et al. (32), UK, Germany	Clinical case study, open-label, non-randomized, non-controlled	66	Tinnitus Handicap Questionnaire (THQ) score	VAS scores were significantly improved: 25.8% mean reduction in tinnitus loudness, 32% mean reduction in tinnitus annoyance (*p* < 0.01 compared to baseline)
			VAS for tinnitus annoyance and loudness	A clinically significant reduction in tinnitus loudness and annoyance was recorded in 59.1 and 72.7% of the patient group, respectively
				THQ scores were significantly improved by an average of 19.4% (*p* < 0.01)
				58.8% of patients experienced a clinically significant reduction in THQ score

In 2012, Tass et al. published a computational study (27) where they illustrated the simplified neuronal model concept of CR anti-kindling and desynchronizing: an algorithm that was also applicable to the concept of non-invasive acoustic CR neuromodulation. In addition, they pointed to some important features of CR: (1) the pitches of the CR tones should be grouped around the patient-specific tinnitus frequency; (2) CR was effective no matter whether the CR stimulus was confined to the deafferentiated region (most effective) or if both the deafferentiated and non-deafferentiated regions were stimulated; and (3) an optimized spacing of the different CR tone pitches was present, neither too narrow, to ideally stimulate distinct subpopulations, nor too wide, to primarily affect the deafferentiated region. Acoustic CR stimulation requires sufficient hearing ability in the deafferentiated region, which can be achieved by hearing aids. The study suggested that acoustic CR neuromodulation may be a reliable method for the control of synchronization and abnormal interactions in affected neuronal populations.

Subsequently, in a single blind RCT (RESET study) (23), Tass et al. examined the safety and effects of acoustic CR neuromodulation in 63 patients with chronic tonal ST, randomly allocated into 5 different treatment arms (G1–G5). They found the intervention to be safe and well tolerated. Acoustic CR neuromodulation resulted in a significant reduction of tinnitus symptoms, as well as VAS loudness and annoyance, improvement in TQ severity levels, reduction in mean TQ scores, and a reduction of tinnitus frequency. Effects gained in 12 weeks of treatment persisted through a 4-week therapy pause. After a long-term extension period (24 weeks), already gained treatment effects were sustained or improved further. Placebo treatment (G5) did not lead to any significant changes in outcome measures. A comparison between groups revealed that CR therapy was more efficacious when used 4–6 h per day as opposed to 1 h per day. After 12 weeks of therapy, tinnitus associated EEG alterations were reversed. Pathologically elevated δ and γ activity were both decreased in the primary and secondary auditory cortex, as well as in frontal brain areas. Tinnitus-related reduction of α activity was reversed, leading to an enhancement in auditory and prefrontal areas. Thus, CR-induced neuronal changes comprised both auditory and non-auditory brain areas.

Based on existing data from 59 of the 63 patients from the RESET study, Adamchic et al. (28) found a significant correlation between the absolute value of the tinnitus pitch (frequency) change induced by acoustic neuromodulation, and absolute changes of VAS loudness and annoyance scores. The study also found that changes of brain synchrony patters, induced by CR neuromodulation, were associated with pitch change. These changes included the decreases in γ power and increases in α activity in distinctive brain regions, as well as alterations in functional connectivity in the γ frequency band between brain areas of this network. Brain areas found to be associated with these changes involved left temporal cortex, right and left frontal areas, the dorsolateral prefrontal region, and the anterior cingulate cortex. The study discovered that acoustic CR neuromodulation induced tinnitus pitch change, with a simultaneous reduction in tinnitus symptoms and positive changes in oscillatory brain activity.

In another evaluation of the data from the RESET study, Adamchic et al. (29) compared the EEG data from 28 patients with bilateral ST with the spontaneous EEG data from healthy controls. The study showed that acoustic CR neuromodulation shifted the abnormal brain activity associated with tinnitus toward physiological levels. Hence, in a group of “good responders” (TQ improvement >12 points), acoustic CR neuromodulation significantly normalized the patient’s brain oscillations and even led to a complete abolishment of pathological power in several brain regions and frequency bands. These changes were significantly correlated with a reduction of tinnitus severity.

In 2013, using the same study population, Silchenko et al. (30) investigated if acoustic CR neuromodulation induced alterations of effective connectivity in the neuronal network underlying tinnitus perception. The effective connectivity in gamma, delta, and alpha frequency bands between brain areas comprising the primary auditory cortex, posterior cingulate cortex, dorsolateral prefrontal areas, and temporal areas were found to be significantly different in ST patients as opposed to healthy controls. When analyzing the types of interactions, they found a significant imbalance of excitation and inhibition in the tinnitus network of brain sources. Acoustic CR therapy significantly altered the strength of these connections so that they approached or even became indistinguishable to the healthy state network structure. Such restoration of effective connectivity was not seen in the group of non-responders.

To further investigate the communicative pathways within the tinnitus network, Adamchic et al. (31) performed a re-analysis of the existing dataset from the RESET study. They found that abnormal CFC^2^ in tinnitus patients might coordinate tinnitus-relevant activity and thus provide effective communication between nodes of the tinnitus network. Reduction of tinnitus severity after acoustic CR stimulation led to a partial normalization of abnormal CFC. Treatment-induced tinnitus pitch change significantly modulated changes in CFC.

In 2015, Hauptmann et al. (26) published a prospective, non-randomized, non-controlled, multicenter, and clinical study with 200 chronic tinnitus patients. TQ “Tinnitus-Beeinträchtigungs-Fragebogen TBF12” (TBF-12) and CGI-I7 were used to study the safety and efficacy of acoustic CR neuromodulation. The treatment was found to be well tolerated and with no adverse events. Acoustic CR neuromodulation caused a statistically and clinically significant decrease in TBF-12 scores as well as in CGI-I7 after 12 months of therapy.

The same year, Williams et al. (32), published a clinical case study, where they described the quantitative treatment outcomes of patients undergoing acoustic CR neuromodulation. In line with the abovementioned clinical study, they showed a statistically and clinically significant improvement in tinnitus symptoms after 22–26 weeks of treatment, measured by VAS scores (loudness and annoyance) and THQ.

A protocol for a double-blind RCT study on the evaluation of acoustic CR neuromodulation was suggested by Hoare et al. (34). Although the behavioral results of the study have not been published, they are available on a trial outcomes website (https://clinicaltrials.gov/ct2/show/results/NCT01541969?sect=X0125#all). The efficacy of acoustic CR neuromodulation was compared to placebo (tinnitus masking), with crossover of the placebo group to receive the proprietary intervention. The study was completed on February 24, 2016. However, deviations from trial protocol and lack of compliance with the manufacturer’s fitting instructions led to doubts about the validity of the results. Hence, results could not be published with evidence, and therefore, it was not included in the present review (35, 36).

## Discussion

This review was performed to provide a view over the existing evidence and use of acoustic CR neuromodulation in the treatment of ST. Overall, relatively few publications were found through the search, and the general evidence level was low except for a single RCT. The technique demands assessment of the specific tinnitus frequency and that the patient has (aided) hearing capacity within the given frequency area. A total of 329 patients with chronic tinnitus were included in basically 3 various study populations. Outcome measures were psycho-acoustic tests and EEG. The majority of the included patients reported reduction of tinnitus that was associated with EEG changes toward normalization. The method intends to counteract abnormal synchronization and connectivity through desynchronizing and anti-kindling effects. More specifically, acoustic CR neuromodulation induced pitch change and partially reversed abnormal CFC in a widespread tinnitus network comprising both auditory and non-auditory brain areas. However, several shortcomings of the studies impede generalization of the obtained results and the neurophysiological basis of the method may also be questioned (see below). Thus, the available evidence is insufficient for confident clinical implementation of acoustic CR neuromodulation as a treatment modality for tinnitus at the present moment.

We only identified 8 original reports about acoustic CR neuromodulation based on 3 different populations, i.e., a maximum of 329 patients. Different scopes of the studies, designs, and outcome parameters impede comparison between them as well as use of mutual quality and bias assessment methods. Moreover, many complicated statistical analyses were applied making it difficult to decipher and interpret the results and overall conclusions. Hence, PRISMA guidelines could not be followed in all aspects.

The same authors have generated most of the studies and articles, using the same populations. Independent replication of the positive pilot results is still lacking.

The proof-of-concept study performed by Tass et al. (23) was a RCT—a study design with the greatest evidential value. However, as also pointed out by Rücker and Antes in their reply (37) to Tass et al., the study had several shortcomings: a small number of participants, use of five treatment arms, and most importantly, the lack of direct comparison between the different treatment arms, which is the main idea of RCT studies. Besides baseline to post treatment comparisons within each treatment arm, no direct comparisons between groups were conducted. Tass et al. argued that the study showed quality features not often encountered in tinnitus research (38). A sample size calculation in order to ensure inclusion of sufficient numbers of participants in the different treatment schedules is recommended prior to such studies: the more subgroups, the more participants. A pilot study is also advantageous, for instance, to determine variation of the outcome measures.

In an additional analysis, different treatment arms were *post hoc* pooled and a comparison between an “effective” versus an “ineffective” group was performed. Such pooling results in loss of information and confounders are introduced due to use of different stimuli within the same group, and a false treatment effect may be the result (37, 39). And finally, the applied statistics were rather complex and unusual (e.g., Euclidean distance).

Clinical series or reports, such as the two clinical case studies (26, 32), are associated with a low level of evidence and the main reason is the absence of a control group. A clinical case study, such as the one conducted by Hauptman et al., is meant to consolidate the results from studies with higher level of evidence, e.g., RESET. With an intention to reinforce previously conducted studies, the risk of bias due to the desire for an effect can be comprised. It should also be mentioned that the use of fee-paying participants in the study by Williams et al. might have affected the results due to selection bias. Despite the low level of evidence, both Hauptman and Williams stated that acoustic CR neuromodulation caused a significant decrease of tinnitus. This trend was seen in different outcome measures.

The clinical case studies differ with respect to inclusion criteria, providing heterogeneous study populations. Future studies should be based on precise inclusion criteria to make them more homogenous. This includes consensus regarding the definition of chronic tinnitus, inclusion of both unilateral and bilateral cases, hearing thresholds, etc. Information about confounding factors such as age, psychiatric disorders, medication, and previously received tinnitus therapy should be addressed. Consensus regarding exclusion criteria is important as well. And finally, use of the same reliable and valid outcome measures in different studies is mandatory for comparing results, e.g., VAS and TQ scores (40, 41).

The fundamental challenge in tinnitus research goes far beyond the abovementioned problems with clinical designs and choice of outcome measures because we still know only little about the underlying pathophysiology. As a consequence hereof, the optimal objective parameters are chosen more or less in the dark, whether EEG or other neuroimaging techniques. In an opinion article by Elgoyhen et al. (42), they critically assessed the results of recent neuroimaging studies of ST. The authors concluded that neuroimaging results are highly variable, but interpretation of available data indicates ST to follow auditory deafferentiation leading to lack of sensory information. Elgoyhen et al. suggested that increased activity in auditory pathways could explain the perception of the sound itself, maybe permitted or facilitated by interactions or communication with non-auditory brain areas. The relevance of cortical map reorganization as the cause of tinnitus was questioned, and the authors pointed out that the apparent distortion of the tonotopic map associated with tinnitus may merely be a compensatory response and not the cause of tinnitus. If so, cortical reorganization becomes less relevant to understanding the mechanisms eliciting the perception of phantom sounds and questions the theoretical basis of acoustic CR neuromodulation. In the light of the suggestions by Elgoyhen et al., the obvious questions are: what do the apparent findings in the reviewed reports represent and what is the background for them? Can listening to series of tones be able to interfere with a complex brain disorder involving several brain structures and functions like tinnitus? Regardless of the physiological mechanisms, the RESET study had limitations in design.

Future studies of acoustic CR neuromodulation should comprise RCTs with strict inclusion and exclusion criteria and using several neuroimaging techniques.

## Conclusion

Acoustic CR neuromodulation has been introduced as a novel treatment for ST. The current evidence level is low, and the assumed underlying physiological mechanisms have been questioned. Therefore, further studies are needed before the method can be recommended as a tinnitus treatment modality.

## Definitions (22)

**Phase reset** (43) – Phase resetting in neurons is when the dynamical behavior of an oscillation is shifted. This occurs when a stimulus perturbs the phase within an oscillatory cycle and a change in period occurs.

**Synchrony** – The relation that exists when things occur at the same time, e.g., when neurons fire simultaneously.

**Desynchronization** – The reverse of or absence of synchrony. Initially synchronized oscillating (fluctuating) systems desynchronize as parameters (existing conditions) changes, or might do so under influence of external stimulation.

**Anti-kindling** – Unlearning of pathologically strong interactions and/or connectivity of neural networks. The intention is to approach or even regain physiological activity.

**Spike timing-dependent plasticity (STDP)** (44) – A process by which neurons continuously regulate the strength of their synaptic connections. The neurons adjust the connection strengths based on the relative timing of a particular neuron’s firing (or spikes).

**Multistability** (45) – Multistability is the characteristic of a system that presents two or more mutually exclusive stable states (attractors) for a given set of parameters or conditions. If a system has multiple coexisting attractors and stimulus is sufficiently strong to cause switching among stable states, it may be said to be multistable.

## Author Contributions

MW: design, data analysis, drafting, acquisition of data final approval, and responsibility for content of manuscript. TO and DL: data analysis, critically revision, final approval, and responsibility for content of manuscript.

## Conflict of Interest Statement

The authors declare that the research was conducted in the absence of any commercial or financial relationships that could be construed as a potential conflict of interest. The reviewer BL and handling editor declared their shared affiliation, and the handling editor states that the process nevertheless met the standards of a fair and objective review.

## Footnotes

^1^Dinces EA. *Tinnitus*. Available from: http://www.uptodate.com/contents/treatment-of-tinnitus?source=machineLearning&search=tinnitus&selectedTitle=2~150&sectionRank=1&anchor=H2#H2

^2^Cross-frequency coupling (CFC) is a phenomenon proposed to coordinate neural dynamics across spatial and temporal scales (33).

## References

[1] Eggermont JJ Roberts LE. The neuroscience of tinnitus. Trends Neurosci (2004) 27(11):676–82. 10.1016/j.tins.2004.08.010 15474168

[2] Eggermont JJ Tass PA. Maladaptive neural synchrony in tinnitus: origin and restoration. Front Neurol (2015) 6:29. 10.3389/fneur.2015.00029 25741316 PMC4330892

[3] Hall DA Láinez MJ Newman CW Sanchez TG Egler M Tennigkeit F Treatment options for subjective tinnitus: self reports from a sample of general practitioners and ENT physicians within Europe and the USA. BMC Health Serv Res (2011) 11:302. 10.1186/1472-6963-11-302 22053947 PMC3227628

[4] Axelsson A Prasher D. Tinnitus induced by occupational and leisure noise. Noise Health (2000) 2(8):47–54. 12689461

[5] Noreña AJ. Revisiting the cochlear and central mechanisms of tinnitus and therapeutic approaches. Audiol Neurotol (2015) 20(1):53–9. 10.1159/000380749 25997584

[6] Baguley D McFerran D Hall D. Tinnitus. Lancet (2013) 382(9904):1600–7. 10.1016/S0140-6736(13)60142-7 23827090

[7] Jastreboff PJ. Phantom auditory perception (tinnitus): mechanisms of generation and perception. Neurosci Res (1990) 8(4):221–54. 10.1016/0168-0102(90)90031-9 2175858

[8] Kemp DT. Stimulated acoustic emissions from within the human auditory system. J Acoust Soc Am (1978) 64(5):1386–91. 10.1121/1.382104 744838

[9] Kaltenbach JA Zhang J Finlayson P. Tinnitus as a plastic phenomenon and its possible neural underpinnings in the dorsal cochlear nucleus. Hear Res (2005) 206(1–2):200–26. 10.1016/j.heares.2005.02.013 16081009

[10] Kaltenbach JA. The dorsal cochlear nucleus as a contributor to tinnitus: mechanisms underlying the induction of hyperactivity. Prog Brain Res (2007) 166:89–106. 10.1016/S0079-6123(07)66009-9 17956775

[11] Levine RA Somatic (craniocervical) tinnitus and the dorsal cochlear nucleus hypothesis. Am J Otolaryngol (1999) 20(6):351–62. 10.1016/S0196-0709(99)90074-1 10609479

[12] Noreña AJ Eggermont JJ. Changes in spontaneous neural activity immediately after an acoustic trauma: implications for neural correlates of tinnitus. Hear Res (2003) 183(1–2):137–53. 10.1016/S0378-5955(03)00225-9 13679145

[13] Kaltenbach JA. Summary of evidence pointing to a role of the dorsal cochlear nucleus in the etiology of tinnitus. Acta Otolaryngol Suppl (2006) (556):20–6. 10.1080/03655230600895309 17114138

[14] Hoare D Sereda M Adjamian P Hall D. Recent technological advances in sound-based approaches to tinnitus treatment: a review of efficacy considered against putative physiological mechanisms. Noise Health (2013) 15(63):107. 10.4103/1463-1741.110292 23571301

[15] Eggermont JJ Roberts LE The neuroscience of tinnitus: understanding abnormal and normal auditory perception. Front Syst Neurosci (2012) 6:53 10.3389/fnsys.2012.00053 22798948 PMC3394370

[16] Hoare DJ Searchfield GD Refaie AE Henry JA. Sound therapy for tinnitus management: practicable options. J Am Acad Audiol (2014) 30(1):62–75. 10.3766/jaaa.25.1.5 24622861

[17] Tass PA Silchenko AN Hauptmann C Barnikol UB Speckmann E-J. Long-lasting desynchronization in rat hippocampal slice induced by coordinated reset stimulation. Phys Rev E Stat Nonlin Soft Matter Phys (2009) 80(1):11902. 10.1103/PhysRevE.80.011902 19658724

[18] Tass PA Qin L Hauptmann C Dovero S Bezard E Boraud T Coordinated reset has sustained aftereffects in parkinsonian monkeys. Ann Neurol (2012) 72(5):816–20. 10.1002/ana.23663 23280797

[19] Adamchic I Hauptmann C Barnikol UB Pawelczyk N Popovych O Barnikol TT Coordinated reset neuromodulation for Parkinson’s disease: proof-of-concept study. Mov Disord (2014) 29(13):1679–84. 10.1002/mds.25923 24976001 PMC4282372

[20] Tass PA. A model of desynchronizing deep brain stimulation with a demand-controlled coordinated reset of neural subpopulations. Biol Cybern (2003) 89(2):81–8. 10.1007/s00422-003-0425-7 12905037

[21] Tass PA Majtanik M. Long-term anti-kindling effects of desynchronizing brain stimulation: a theoretical study. Biol Cybern (2006) 94(1):58–66. 10.1007/s00422-005-0028-6 16284784

[22] Popovych OV Tass PA Control of abnormal synchronization in neurological disorders. Front Neurol (2014) 5:268 10.3389/fneur.2014.00268 25566174 PMC4267271

[23] Tass PA Adamchic I Freund HJ Von Stackelberg T Hauptmann C. Counteracting tinnitus by acoustic coordinated reset neuromodulation. Restor Neurol Neurosci (2012) 30(2):137–59. 10.3233/RNN-2012-110218 22414611

[24] Lysyansky B Popovych OV Tass PA Desynchronizing anti-resonance effect of m: n ON–OFF coordinated reset stimulation. J Neural Eng (2011) 8(3):36019 10.1088/1741-2560/8/3/036019 21555848

[25] Chittka L Brockmann A Perception space—the final Frontier. PLoS Biol (2005) 3(4):e137 10.1371/journal.pbio.0030137 15819608 PMC1074815

[26] Hauptmann C Ströbel A Williams M Patel N Wurzer H von Stackelberg T Acoustic coordinated reset neuromodulation in a real life patient population with chronic tonal tinnitus. Biomed Res Int (2015) 2015:569052. 10.1155/2015/569052 26568958 PMC4629059

[27] Tass PA Popovych OV. Unlearning tinnitus-related cerebral synchrony with acoustic coordinated reset stimulation: theoretical concept and modelling. Biol Cybern (2012) 106(1):27–36. 10.1007/s00422-012-0479-5 22350536

[28] Adamchic I Hauptmann C Tass PA Changes of oscillatory activity in pitch processing network and related tinnitus relief induced by acoustic CR neuromodulation. Front Syst Neurosci (2012) 6:18 10.3389/fnsys.2012.00018 22493570 PMC3319974

[29] Adamchic I Toth T Hauptmann C Tass PA. Reversing pathologically increased EEG power by acoustic coordinated reset neuromodulation. Hum Brain Mapp (2014) 35(5):2099–118. 10.1002/hbm.22314 23907785 PMC4216412

[30] Silchenko AN Adamchic I Hauptmann C Tass PA. Impact of acoustic coordinated reset neuromodulation on effective connectivity in a neural network of phantom sound. Neuroimage (2013) 77:133–47. 10.1016/j.neuroimage.2013.03.013 23528923

[31] Adamchic I Langguth B Hauptmann C Tass PA Abnormal cross-frequency coupling in the tinnitus network. Front Neurosci (2014) 8:284 10.3389/fnins.2014.00284 25309309 PMC4174755

[32] Williams M Hauptmann C Nitesh P. Acoustic CR neuromodulation therapy for subjective tonal tinnitus: a review of clinical outcomes in an independent audiology practice setting. Front Neurol (2015) 6:54. 10.3389/fneur.2015.00054 25838816 PMC4362296

[33] Aru J Aru J Priesemann V Wibral M Lana L Pipa G Untangling cross-frequency coupling in neuroscience. Curr Opin Neurobiol (2015) 31:51–61. 10.1016/j.conb.2014.08.002 25212583

[34] Hoare DJ Pierzycki RH Thomas H McAlpine D Hall DA Evaluation of the acoustic coordinated reset (CR^®^) neuromodulation therapy for tinnitus: study protocol for a double-blind randomized placebo-controlled trial. Trials (2013) 14(1):207 10.1186/1745-6215-14-207 23842505 PMC3710266

[35] Hoare DJ Pierzycki RH Thomas H Hall DA Evaluation of the CR Neuromodulation Treatment for Tinnitus – Study Results – ClinicalTrials. (2015). Available from: https://clinicaltrials.gov/ct2/show/results/NCT01541969?sect=X01256#all

[36] Hall DA Hoare DJ Pierzycki RH 59257caf5076ef0653477c5964fa1512e468b0e8. (2015). Available from: www.hearing.nihr.ac.uk; http://www.hearing.nihr.ac.uk/research/a-systematic-evaluation-of-the-acoustic-cr-neuromodulation-treatment-for-ti/

[37] Rücker G Antes G Reply to Tass et al. on “Counteracting tinnitus by acoustic coordinated reset neuromodulation”. Restor Neurol Neurosci (2012) 30(2); *Restor Neurol Neurosci* (2013) 31(3):235–7. 10.3233/RNN-121123 23328229

[38] Tass PA Adamchic I Freund H-J von Stackelberg T Hauptmann C Rebuttal to reply by G. Rücker and G. Antes on Tass et al. “Counteracting tinnitus by acoustic coordinated reset neuromodulation”. Restor Neurol Neurosci (2012) 30(2); *Restor Neurol Neurosci* (2013) 31(3):235–7. 10.3233/RNN-121123 23314005

[39] Senn S Julious S. Measurement in clinical trials: a neglected issue for statisticians? Stat Med (2009) 28(26):3189–209. 10.1002/sim.3603 19455540

[40] Adamchic I Langguth B. Psychometric evaluation of Visual Analog Scale for the assessment of chronic tinnitus. Am J Audiol (2012) 21:215–26. 10.1044/1059-0889(2012/12-0010)impairment 22846637

[41] Adamchic I Tass PA Langguth B Hauptmann C Koller M Schecklmann M Linking the Tinnitus Questionnaire and the subjective Clinical Global Impression: which differences are clinically important? Health Qual Life Outcomes (2012) 10(1):79. 10.1186/1477-7525-10-79 22781703 PMC3487915

[42] Elgoyhen AB Langguth B De Ridder D Vanneste S. Tinnitus: perspectives from human neuroimaging. Nat Rev Neurosci (2015) 16(10):632–42. 10.1038/nrn4003 26373470

[43] Krogh-Madsen T Butera R Ermentrout GB Glass L Chapter 2: Phase resetting neural oscillators: topological theory versus the realworld. In: Schultheiss NW Prinz AA Butera RJ, editors. Phase Response Curves in Neuroscience, Theory, Experience End Analysis. Vol. 6 New York: Springer New York (2012).

[44] Markram H Gerstner W Sjöström PJ Spike-timing-dependent plasticity: a comprehensive overview. Front Synaptic Neurosci (2012) 4:2 10.3389/fnsyn.2012.00002 22807913 PMC3395004

[45] Kelso JAS. Multistability and metastability: understanding dynamic coordination in the brain. Philos Trans R Soc Lond B Biol Sci (2012) 367:906–18. 10.1098/rstb.2011.0351 22371613 PMC3282307

